# PTMA, a new identified autoantigen for oral submucous fibrosis, regulates oral submucous fibroblast proliferation and extracellular matrix

**DOI:** 10.18632/oncotarget.20419

**Published:** 2017-08-24

**Authors:** Jie Wang, Jialing You, Lekai Wang, Huimin Wang, Tian Tian, Wenjin Wang, Lina Jia, Canhua Jiang

**Affiliations:** ^1^ Department of Oral and Maxillofacial Surgery, Xiangya Hospital, Central South University, Changsha 410078, China; ^2^ Department of Immunology, Xiangya School of Medicine, Central South University, Changsha 410078, China

**Keywords:** oral submucous fibrosis (OSF), OSF-associated autoantigens, PTMA, fibroblast, extracellular matrix (ECM)

## Abstract

Oral submucous fibrosis (OSF) is a chronic, insidious disease. The presence of autoantibodies in sera of OSF patients is the most characteristic and direct evidence of OSF being an autoimmune disease. To identify the specific autoantigens which could contribute to antibody production, the Human Proteome Microarrays composed of 19000 full-length unique proteins were employed. 45 proteins correlated with OSF were identified. To validate these results, we used ELISA to validate 28 OSF-associated autoantigens in extended samples. 8 autoantigens were positive in OSF serum with high frequency compared to the healthy controls. Moreover, the mRNA expression of 8 candidates was up-regulated in OSF oral submucous tissues; among them, the protein level of PTMA, the one with the highest positive frequency, was also increased. Through searching the Bioinformatics Public Database and performing the Spearman’s rank correlation analysis, we observed that PTMA was positively correlated with fibrosis-related TGFβ1 and SMAD4, the downstream gene of TGFβ1. In TGFβ1-induced fibrosis model of primary human oral submucous fibroblast, PTMA knockdown reversed TGFβ1-induced fibrosis process through inhibiting the cell viability and proliferation of fibroblast, reducing the protein levels of PTMA, Collagen I, α-SMA and MMP9 and increasing the protein levels of SMAD4. In contrast, PTMA overexpression enhanced TGFβ1-induced fibrosis process. Taken together, PTMA is involved in TGFβ1-induced fibrosis in the primary human submucous fibroblast by regulating the expression of ECM-related markers and the downstream genes of TGFβ1. In conclusion, PTMA presents an essential autoantigen during OSF process; targeting PTMA might be a promising strategy for OSF treatment.

## INTRODUCTION

Oral submucous fibrosis (OSF) is a chronic disease of the oral cavity characterized by inflammation and progressive mucosal fibrosis. These reactions may be the result of either direct stimulation from exogenous antigens like areca alkaloids or by changes in tissue antigenicity that may lead to an autoimmune response [1-5]. Cytokines and growth factors produced by the inflammatory cells within the OSF tissues may promote OSF by inducing the proliferation of fibroblasts, up-regulating the collagen synthesis and down-regulating the collagenase production [6]. OSF fibroblasts have been shown to produce the collagen with more stable structure such as collagen type I trimer [7] and secrete more lysyl oxidase which causes an increase in collagen cross-linkages [8, 9].

In addition to over-proliferation and altered production of the indicated markers, increase in serum levels of immunoglobulins [10, 11] and in circulating immune complexes and their immunoglobulin contents [12, 13] have been reported in OSF patients. The defects in cellular immunity have also been observed in patients with OSF [12]. Furthermore, the high incidence of autoantibodies including antinuclear (ANA), antismooth muscle (SMA), antigastric parietal cell (GPCA), antithyroid microsomal (TMA), and antireticulin antibodies have been observed in patients with OSF [10]. A genetic predisposition, involving the HLA antigens A10, DR3, DR7, and haplotypic pairs A10/DR3, B8/DR3, and A10/B8 has also been demonstrated in OSF patients [10].

The presence of autoantibodies in sera of OSF patients is the most characteristic and direct evidence to support that OSF may be an autoimmune disease. Chiang et al. [14] found that the frequencies of presence of serum ANA, SMA, and GPCA in OSF patients were significantly higher than those in healthy control subjects, which were associated with daily consumption of areca quid (AQ). Interestingly, altered autoantigens released from AQ ingredients-damaged cells may induce autoantibody production. Higher frequencies of specific HLA-DR antigens in OSF patients may also help autoantibody production [14]. Besides, Haque et al. [15] reported the increased CD4 and HLA-DR-positive cells in OSF tissues, which suggests that most lymphocytes were activated, indicating an ongoing cellular immune response leading to a possible imbalance of immunoregulation and alteration in local tissue architecture. However, specific autoantigens in OSF patients have yet to be found.

The recently developed functional protein microarrays were designed to survey thousands of potential antigens in a single experiment and have facilitated rapid and cost effective identification of novel biomarkers [16-18]. Here, we employed a recently developed Human Proteome Microarrays to screen for novel OSF-specific biomarkers. The resulting OSF-associated candidate autoantigens were then used for additional validation using ELISA, QPCR and Western blot assays to demonstrate their utility in OSF. We then further checked Bioinformatics Public Database for the potential association between PTMA, an autoantigen with the highest positive rate, and fibrosis-related key factors, TGFβ1 and SMAD4. Using TGFβ1-induced cell fibrosis model, we investigated the detailed functions of PTMA in TGFβ1-induced oral submucous fibroblast proliferation and extracellular matrix (ECM). Taken together, we revealed that PTMA is involved in TGFβ1-induced fibrosis by regulating the expression of TGFβ1 downstream gene, and present a novel promising strategy for OSF treatment.

## RESULTS

### Identification of OSF-associated autoantigens using the human proteome microarrays

**Figure 1:**
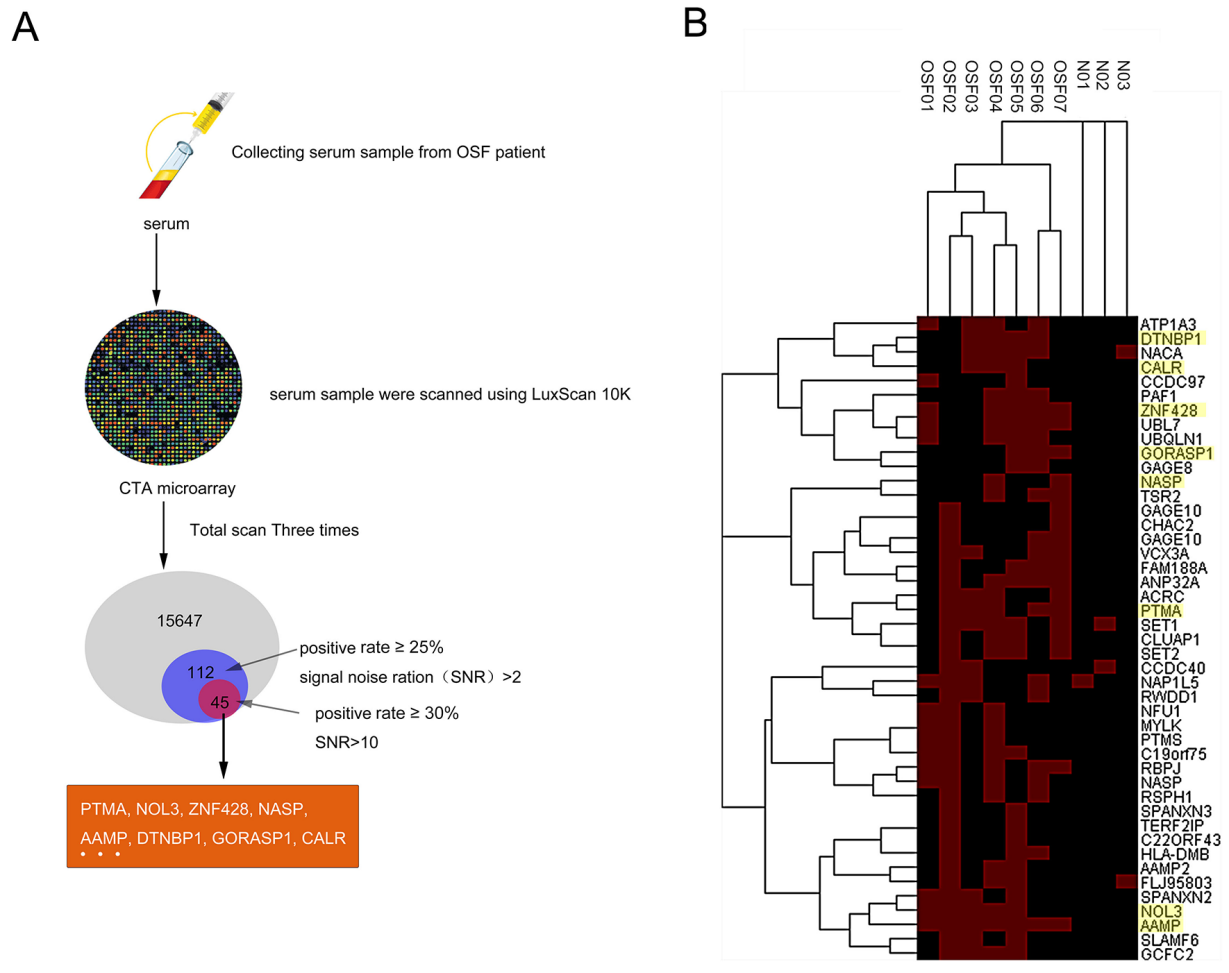
Identification of OSF-associated autoantigens using the human proteome microarrays **(A)** The flow chart of OSF-related autoantigens selecting using HuProt™ microarray scanning. **(B)** Hierarchical clustering of obtained 45 proteins in OSF and healthy control serum.

To globally identify the OSF-associated autoantigens, we employed the human proteome microarrays containing about 19,000 human proteins to perform serum profiling of samples collected from 7 OSF patients (OSF01-07) and 3 healthy controls (N01-03). Because the human proteins on the microarrays were expressed and purified from yeast as N-terminally tagged GST fusion proteins, the quality of the microarray was able to be evaluated by applying the anti-GST antibody. All the sera samples were scanned using LuxScan 10K. A total of 15647 proteins were detectable, of which 112 hits obtained (positive rate ≥25%, signal noise ratio (SNR)>2), and 45 hits obtained after rescreening (positive rate ≥30%, signal noise ratio (SNR)>10), including PTMA, NOL3, ZNF428, NASP, AAMP, DTNBP1, GORASP1, CALR and so on (Figure 1A and 1B). The hierarchical clustering of the identified 45 autoantigens proteins in OSF and control serum was shown in Figure 1B. Detail information for identified OSF-associated autoantigens is shown in Supplementary Table 1.

### ELISA for OSF-associated autoantigens in extended samples

**Figure 2:**
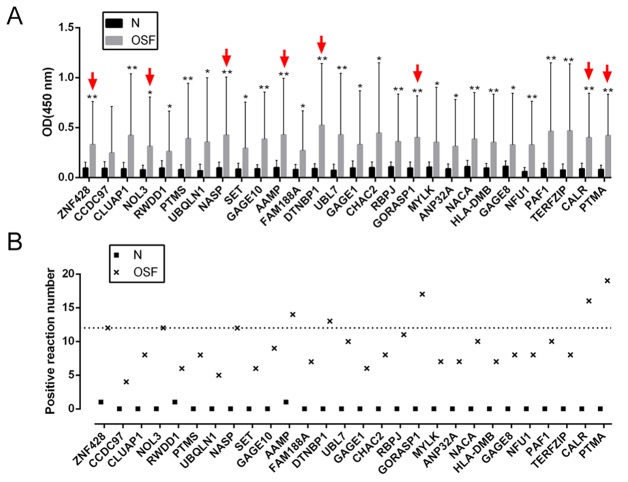
ELISA for OSF-associated autoantigens in extended samples **(A)** 28 proteins of the 45 selected OSF-associated autoantigens were expressed and purified for ELISA assays. The average OD of OSF patient serum group (n=30) and normal healthy control group (n=20) were detected by a microplate reader. The data are presented as mean ± SD of three independent experiments. *P<0.05, **P<0.01, vs healthy control group. **(B)** The positive frequency of the 28 autoantigen candidates in 30 cases of OSF patients, compared to the 20 healthy controls.

To validate the potential autoantigens identified on the human proteome microarrays, and to determine their respective sensitivities and specificities for OSF diagnosis, we prokaryotic expressed and purified 28 of the 45 candidates for further ELISA assays (Supplementary Figure 1). To determine the positive rate of the antibodies to the 28 candidates in sera from 30 OSF patients and 20 healthy control, we performed ELISA assays (Table 1, Figure 2A and 2B). Results showed that the OD450 values of all the 28 OSF samples were significantly higher than those of the healthy control samples (Figure 2A). Using the *A*_450_ > 0.2 as the cutoff for positive hits, we found that the difference of positive rates between the OSF patients and healthy controls for 25 of 28 autoantigens was statistically significant (*P* < 0.05, Figure 2B, Table 1). Here, eight autoantigens with the highest positive rates were selected for further assays: PTMA, NOL3, ZNF428, NASP, AAMP, DTNBP1, GORASP1 and CALR.

**Table 1:** Positive numbers and rates for 28 antigens in OSF and Healthy serum samples

Autoantigens	Sensitivity in groups		p-Value
	Healthy	OSF	
PTMA	0/20	19/30	<0.001
	0.00%	63.33%	
CALR	0/20	16/30	<0.001
	0.00%	53.33%	
GORASP1	0/20	17/30	<0.001
	0.00%	56.67%	
AAMP	1/20	14/30	0.002
	5.00%	46.67%	
DTNBP1	0/20	13/30	0.001
	0.00%	43.33%	
ZNF428	1/20	12/30	0.006
	5.00%	40.00%	
NASP	0/20	12/30	0.001
	0.00%	40.00%	
NOL3	0/20	12/30	0.001
	0.00%	40.00%	
CLUAP1	0/20	8/30	0.012
	0.00%	26.67%	
RBPJ	0/20	11/30	0.002
	0.00%	36.67%	
UBL7	0/20	10/30	0.004
	0.00%	33.33%	
PAF1	0/20	10/30	0.004
	0.00%	33.33%	
NACA	0/20	10/30	0.004
	0.00%	33.33%	
GAGE10	0/20	9/30	0.007
	0.00%	30.00%	
CHAC2	0/20	8/30	0.012
	0.00%	26.67%	
PTMS	0/20	8/30	0.012
	0.00%	26.67%	
GAGE8	0/20	8/30	0.012
	0.00%	26.67%	
NFU1	0/20	8/30	0.012
	0.00%	26.67%	
TERFZIP	0/20	8/30	0.012
	0.00%	26.67%	
FAM188A	0/20	7/30	0.02
	0.00%	23.33%	
HLA-DMB	0/20	7/30	0.02
	0.00%	23.33%	
MYLK	0/20	7/30	0.02
	0.00%	23.33%	
ANP32A	0/20	7/30	0.02
	0.00%	23.33%	
GAGE1	0/20	6/30	0.033
	0.00%	20.00%	
SET	0/20	6/30	0.033
	0.00%	20.00%	
RWDD1	1/20	6/30	0.134
	5.00%	20.00%	
UBQLN1	0/20	5/30	0.054
	0.00%	16.67%	
CCDC97	0/1	4/30	0.089
	0.00%	13.33%	

### Expression of OSF-associated autoantigens in oral submucosa of OSF patients

**Figure 3:**
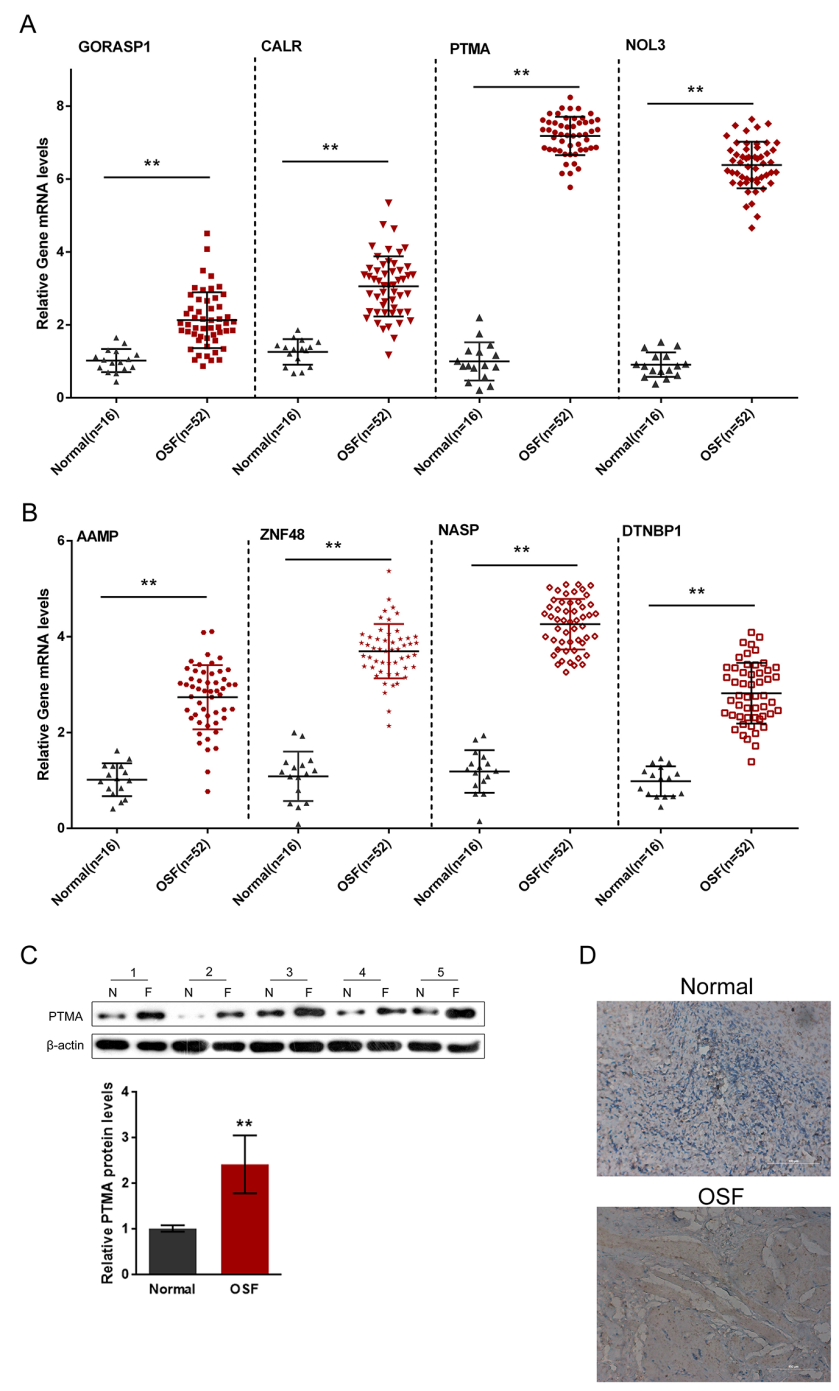
Expression of OSF-associated autoantigens in oral submucosa of OSF patients **(A** and **B)** The mRNA expressions of PTMA, NOL3, ZNF428, NASP, AAMP, DTNBP1, GORASP1 and CALR in OSF submucous tissues were determined using QPCR assays, compared to the normal oral submucosa tissues. **(C** and **D)** The protein expression of PTMA in OSF submucous tissues were determined using Western blot assays and immunochemistry, compared to the normal oral submucosa tissues. The data are presented as mean ± SD of three independent experiments. **P<0.01, vs normal group.

A total of 16 healthy control oral submucosa samples and 52 oral submucosa samples from patients with OSF were collected for further QPCR assays. The mRNA expression of the indicated eight autoantigens in OSF and healthy control oral submucosa tissues were then evaluated. Results showed that the mRNA expressions of all the eight autoantigens were significantly up-regulated, compared to those of the healthy controls (Figure 3A and 3B), which is consistent with the results from the human proteome microarrays scanning. Of the eight autoantigens, the positive rate of PTMA in OSF oral submucosa tissues was the highest (63.33%, P < 0.001); we further evaluated the protein level of PTMA in OSF and healthy oral submucosa tissues using Western blot assays and immunochemistry. Western blot results showed that in five unpaired randomly selected OSF and healthy samples, the protein levels of PTMA were significantly increased in all the five OSF oral submucosa tissues, compared to those of the healthy controls (Figure 3C). In addition, the immunochemistry results also showed a significant increase of PTMA in OSF oral submucosa tissues (Figure 3D).

### PTMA is associated with fibrosis signaling pathway

**Figure 4:**
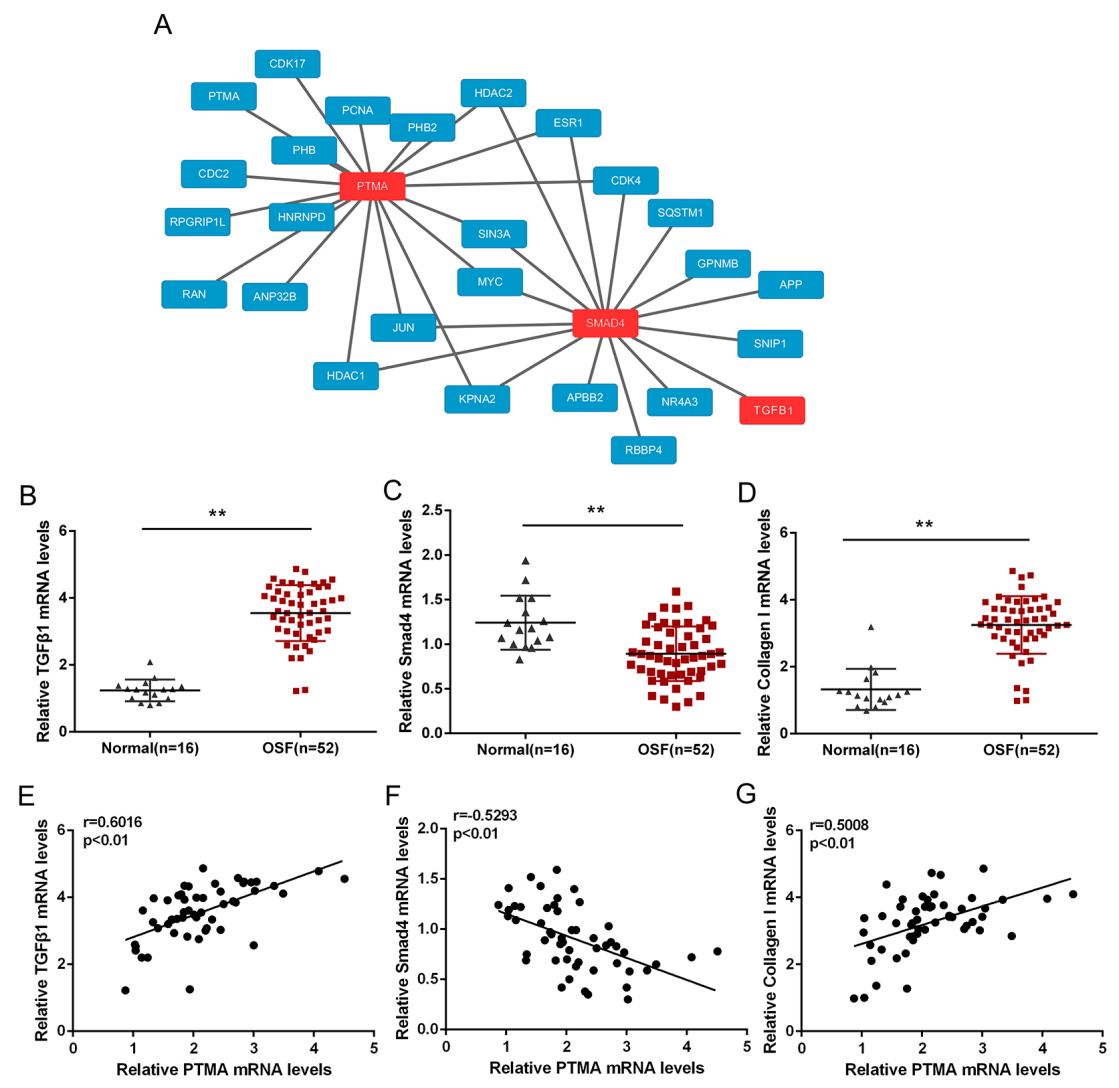
PTMA is associated with fibrosis signaling pathway **(A)** The protein-protein interactionbetween PTMA and fibrosis-related factors, predicted by the Bioinformatics Public Database. **(B** and **D)** The mRNA expression of TGFβ1, Smad4 and Collagen I in OSF and normal healthy control tissues, as determined using QPCR assays. The data are presented as mean ± SD of three independent experiments. **P<0.01, vs normal oral submucosa group. **(E** and **G)** The correlation between PTMA and TGFβ1, PTMA and SMAD, PTMA and Collagen I in OSF oral submucosa tissues, as analyzed using Spearman’s rank correlation analysis.

We have already revealed that PTMA was highly expressed in OSF tissues; to investigate its detailed role in OSF pathology, we searched the bioinformatics public database to see whether there was interaction between PTMA and fibroblast-related proteins, TGFβ1 and SMAD4 (Figure 4A). We found that PTMA, TGFβ1 and SMAD4 shares several transcriptional factors, including MYC, JUN and SIN3A (Figure 4A). To investigate the role of their interactions in OSF pathology, we evaluated the expression of TGFβ1, SMAD4 and Collagen I in 52 OSF tissues and 16 healthy controls using QPCR. Results showed that TGFβ1 and Collagen I mRNA expression was significantly up-regulated in OSF tissues, whereas SMAD4 mRNA was down-regulated in OSF tissues, compared to healthy controls (Figure 4B–4D). Further, we performed Spearman’s rank correlation analysis to analyze the correlation of PTMA and TGFβ1, SMAD4 and Collagen I. Results showed that PTMA mRNA was positively correlated to TGFβ1 and Collagen I, respectively, inversely correlated to SMAD4, suggesting the potential interaction among them and the potential roles of these interactions in OSF.

### TGFβ1 effectively induced fibrosis process in primary human oral submucosa fibroblasts

**Figure 5:**
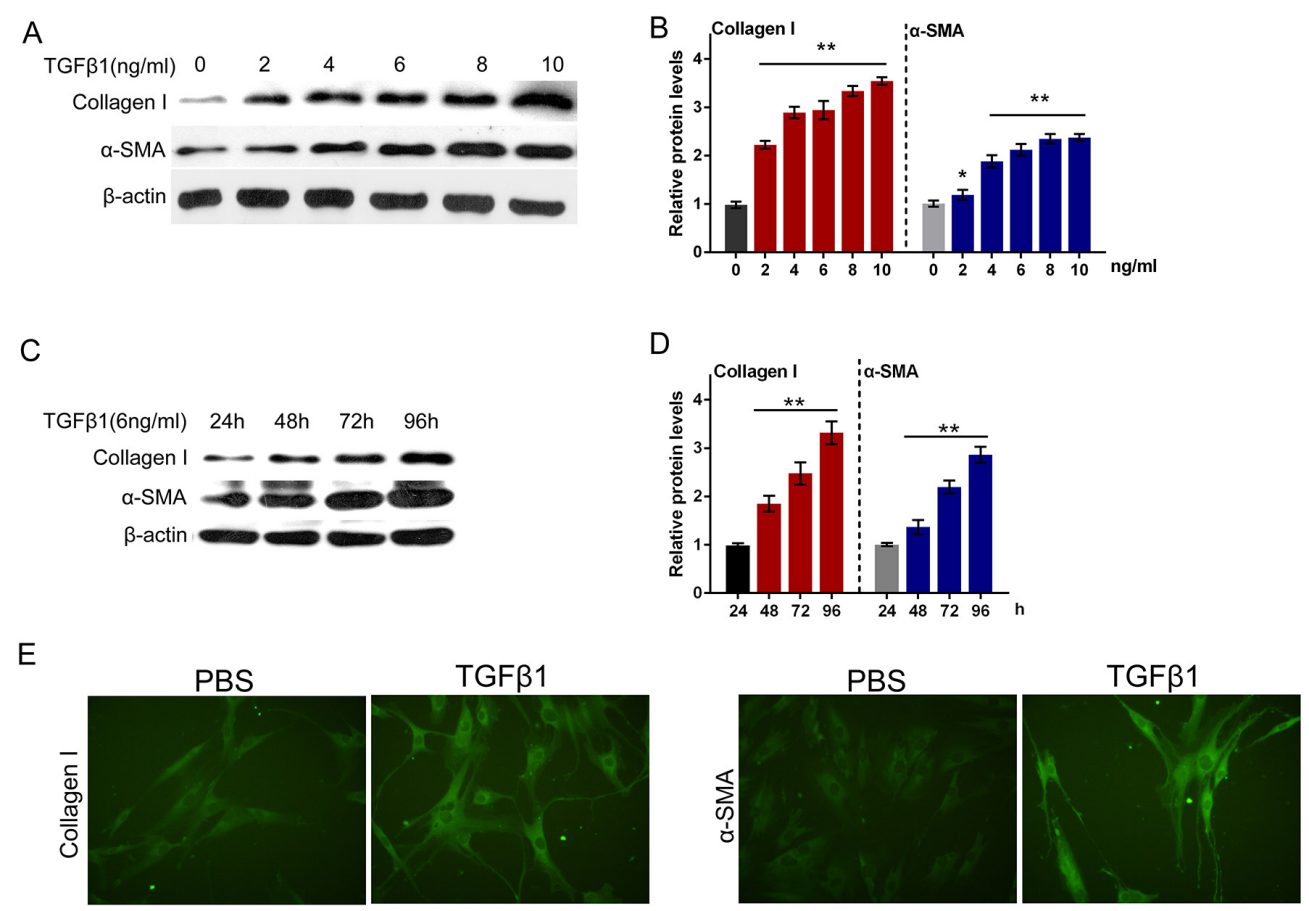
TGFβ1 effectively induced fibrosis process in primary human oral submucosa fibroblasts **(A** and **B)** The protein levels of Collagen I and α-SMA under a series of doses of TGFβ1 treatment (0, 2, 4, 6, 8, 10 ng/ml), as determined using Western blot assays. **(C** and **D)** The protein levels of Collagen I and α-SMA under 6 ng/ml TGFβ1 treatment for 24, 48, 72, 96 h, as determined using Western blot assays. The data are presented as mean ± SD of three independent experiments. **P<0.01, vs 0 ng/ml TGFβ1 group. **(E)** The immunofluorescence assays were performed to validate Collagen I and α-SMA expression of oral submucosa fibroblasts response to TGFβ1 (6 ng/ml for 48 h).

It was well documented that TGFβ1 is a potently fibrogenic growth factor, and the TGFβ1 treatment is a classic method for building fibrosis model [19, 20]. Next, we isolated the normal primary human oral submucosa fibroblasts and constructed fibroblast fibrosis model by a series of doses of TGFβ1 treatment (0, 2, 4, 6, 8, 10 ng/ml). By monitoring the protein levels of Collagen I and α-SMA, the markers of ECM, we evaluated the fibrosis degree of primary human oral submucosa fibroblasts. As shown in Figure 5A and 5B, the protein levels of both Collagen I and α-SMA increased along with the TGFβ1 dose, indicating that TGFβ1 induced fibrosis process in primary human oral submucosa fibroblasts (Figure 5A and 5B). We then treated the primary human oral submucosa fibroblasts with 6 ng/ml TGFβ1 for 24, 48, 72, 96 h, and monitored the protein levels of Collagen I and α-SMA. Results showed that under 6 ng/ml TGFβ1 stimulation, Collagen I and α-SMA increased in a time-dependent manner (Figure 5C and 5D). Further, we performed immunofluorescence assays to validate Collagen I and α-SMA expression of oral submucosa fibroblasts. As exhibited in Figure 5E, the fluorescence intensity in the TGFβ1-stimulated fibroblasts was obviously stronger than the control group, indicating that TGFβ1 effectively induced fibrosis process in primary human oral submucosa fibroblasts (Figure 5E).

### Role of PTMA in TGFβ1-induced fibrosis process

**Figure 6:**
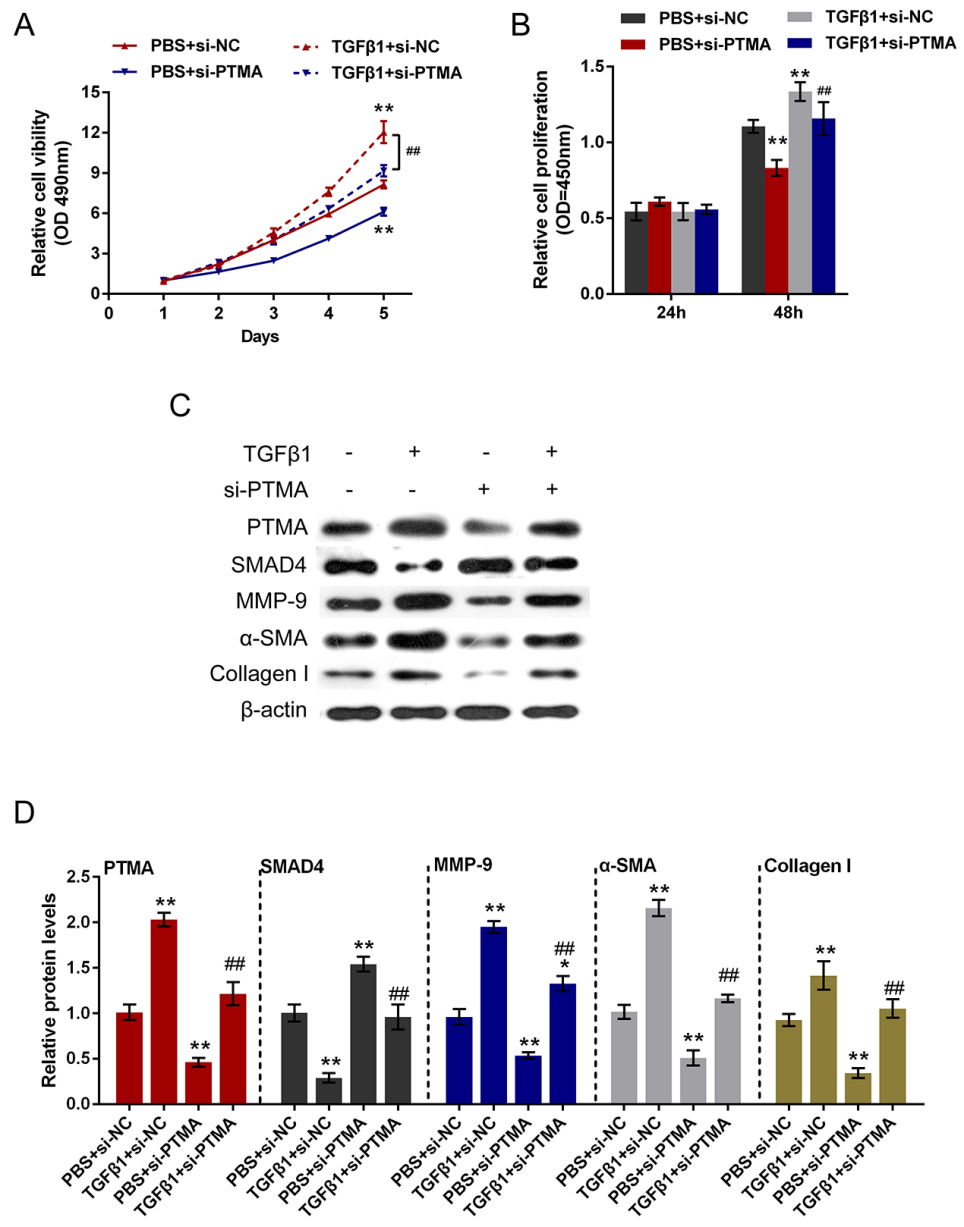
Role of si-PTMA in TGFβ1-induced fibrosis process **(A** and **B)** The primary human oral submucosa fibroblasts were transfected with si-PTMA with the absence or presence of TGFβ1 treatment; the cell viability and proliferation of the fibroblast were then determined using CCK-8 and BrdU assays. **(C** and **D)** The primary human oral submucosa fibroblasts were transfected with si-PTMA with the absence or presence of TGFβ1 treatment; the protein levels of PTMA, SMAD4, MMP-9, α-SMA and Collagen I were then determined using Western blot assays. The data are presented as mean ± SD of three independent experiments. **P<0.01, vs PBS + si-NC group, ## *P*<0.01, vs TGFβ1 + si-NC group

**Figure 7:**
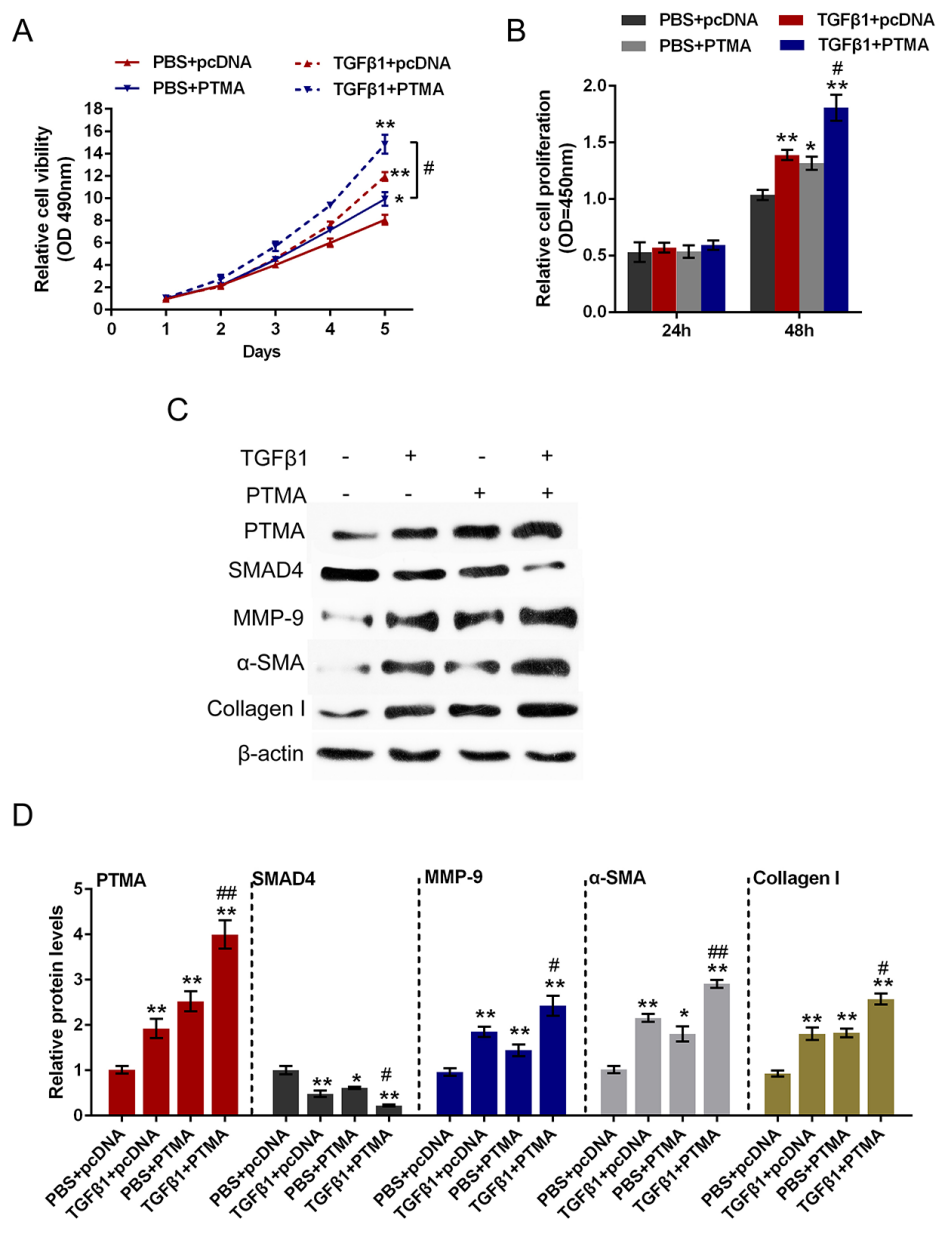
Role of PTMA overexpression in TGFβ1-induced fibrosis process **(A** and **B)** The primary human oral submucosa fibroblasts were transfected with pcDNA-PTMA with the absence or presence of TGFβ1 treatment; the cell viability and proliferation of the fibroblast were then determined using CCK-8 and BrdU assays. **(C** and **D)** The primary human oral submucosa fibroblasts were transfected with pcDNA-PTMA with the absence or presence of TGFβ1 treatment; the protein levels of PTMA, SMAD4, MMP-9, α-SMA and Collagen I were then determined using Western blot assays. The data are presented as mean ± SD of three independent experiments. **P<0.01, vs PBS + pcDNA group, ## *P*<0.01, vs TGFβ1 + TPMA group.

To investigate the detailed function of PTMA in TGFβ1-induced fibrosis process, we achieved PTMA knockdown or overexpression by si-PTMA or pcDNA-PTMA transfection. The cell viability and proliferation of the primary human oral submucosa fibroblasts with the presence or absence of TGFβ1 stimulation using CCK-8 and BrdU assays. Results showed that TGFβ1 stimulation significantly promoted the cell viability and proliferation of fibroblast; PTMA knockdown significantly suppressed the cell viability and proliferation; the promotive effect of TGFβ1 on the cell viability and proliferation could be partially reversed by PTMA knockdown (Figure 6A and 6B). In contrast, PTMA overexpression enhanced the effect of TGFβ1 on cell viability and proliferation (Figure 7A and 7B).

As we mentioned, PTMA was correlated with TGFβ1, SMAD4 and Collagen I in OSF tissues. Here we further monitored the protein levels of PTMA, SMAD4, Collagen I, α-SMA, MMP9 (a downstream factor of SMAD4) with the presence or absence of TGFβ1 stimulation using Western blot assays. Results showed that TGFβ1 stimulation increased the protein levels of PTMA, MMP9, Collagen I and α-SMA, suppressed SMAD4 protein; si-PTMA transfection significantly suppressed PTMA, MMP9, Collagen I and α-SMA protein, whereas increased SMAD4 protein; moreover, the effect of TGFβ1 on the indicated proteins could be partially reversed by PTMA knockdown. On the contrary, PTMA overexpression further enhanced PTMA, MMP9, Collagen I and α-SMA protein expression, whereas reduced SMAD4 protein expression. (Figure 6C and 6D, Figure 7C and 7D). These data indicated that PTMA is involved in TGFβ1-induced fibrosis in the primary human submucous fibroblast by regulating the expression of ECM-related markers and the downstream genes of TGFβ1.

## DISCUSSION

In the present study, we employed the Human Proteome Microarrays to identify OSF-associated autoantigens; of the 45 candidate autoantigens, 28 OSF immunogen-associated proteins were further purified, the antibodies of the 28 antigens in OSF and healthy serum samples were then detected. The mRNA expressions of 8 candidates with higher positive frequency in serum were also significantly up-regulated in OSF oral submucosa tissues, and the protein level of PTMA, the autoantigen with the highest positive frequency, was also up-regulated in OSF submucosa tissues. Further, we found the potential association between PTMA and TGFβ1 and SMAD4, respectively. By establishing TGFβ1-induced oral submucosa fibroblast fibrosis model, we evaluated the function and the underlying mechanism of PTMA in OSF. Taken together, PTMA is involved in TGFβ1-induced fibrosis by regulating the expression of TGFβ1 downstream genes.

OSF is characterized by inflammation and progressive mucosai fibrosis. Direct stimulation from exogenous antigens like areca alkaloids or changes in tissue antigenicity that may lead to an autoimmune response could induce these alternations. However, the mechanisms for the induction and production of various autoantibodies in the sera of OSF patients are still not clear. Chiang et al. revealed that altered autoantigens released from AQ ingredients-damaged cells may induce autoantibody production; higher frequencies of specific HLA-DR antigens in OSF patients may also help autoantibody production [14]. In the present study, we employed the Human Proteome Microarrays to identify OSF-associated autoantigens. After three times of total scan, 45 hits were obtained (positive points ≥6/20, signal noise ratio (SNR)>10), including PTMA, NOL3,ZNF428, NASP, AAMP, DTNBP1, GORASP1, CALR and so on. Further, ELISA assays verified that the antibodies of 28 OSF-associated autoantigens were positive in OSF patients sera, and the positive frequency was significantly higher than that of the healthy controls, indicating the potential roles of these autoantigens in OSF pathology. Moreover, QPCR verified that the mRNA expression of the 8 autogentigens with higher frequency was significantly up-regulated compared to the healthy controls in oral submucosa tissues, which was consistent with the results of proteome microarray and ELISA assays.

Of the selected autoantigens, PTMA obtained the highest positive frequency; further results from Western blot assays showed that PTMA protein in OSF submucous tissues was increased compared to healthy controls. To investigate the potential role of PTMA in OSF pathology, especially in fibrosis process, we searched the bioinformatics public database to see whether PTMA was associated with fibrosis-related factors, TGFβ1 and SMAD4. Results showed that PTMA was associated with TGFβ1 and SMAD4 through transcriptional proteins MYC, JUN and SIN3A. TGF-β plays a specific role in regulation of ECM by regulating the synthesis and degradation of specific ECM components. Upregulation of TGF-β has been demonstrated in OSF and has been proposed to play a pivotal role in the aetiopathology [21, 22]. Aberrant expression of this growth factor has also been reported in keloids, atherosclerosis, pulmonary and liver fibrosis and scleroderma [23]. Imatinib is thought to affect fibrotic pathways by selectively interferring with TGFβ signaling pathways [24]. Imatinib has been used as an antifibrotic drug for experimental treatment of scleroderma [25] and may have a role in placebo-controlled studies in OSF. Here, we observed the high expression of TGFβ1 and Collagen I mRNA in OSF tissues, indicating the activation of TGFβ1-associated fibrosis molecular signaling pathways. Moreover, PTMA was positively correlated with TGFβ1 and Collagen I, inversely correlated with SMAD4 in OSF tissues, suggesting that PTMA might affect the fibrosis process during OSF through interaction with TGFβ1 and regulating ECM-related factors.

What is the detailed function of PTMA in OSF process? How does PTMA exert its function? To investigate these unsolved problems, we established fibrosis model in primary human submucous fibroblast by TGFβ1 stimulation, as verified by up-regulated expression of ECM markers, Collagen I and α-SMA. In TGFβ1-induced fibrosis model of primary human submucous fibroblast, PTMA expression was increased. After PTMA knockdown, TGFβ1-induced cell viability and proliferation of submucous fibroblasts were significantly suppressed. PTMA knockdown also significantly reduced the protein levels of the ECM markers, Collagen I and α-SMA, and cell proliferation-related factors, MMP9, increased the protein levels of the downstream factor of TGFβ1, SMAD4, an inhibitor of MMP9. Moreover, the effects of TGFβ1 on the indicated proteins could be partially reversed by PTMA knockdown. In contrast, PTMA overexpression enhanced the effect of TGFβ1 on cell proliferation and the indicated proteins. These data indicated that the positive role of PTMA in the fibrosis process of the primary human submucous fibroblast and OSF process.

Taken together, we revealed the high frequency of autoantigen PTMA in OSF submucous tissues. PTMA mRNA and protein level is up-regulated in OSF submucous tissues. Moreover, PTMA is involved in TGFβ1-induced fibrosis in the primary human submucous fibroblast by regulating the expression of ECM-related markers and the downstream genes of TGFβ1. In conclusion, PTMA might present an essential autoantigen during OSF process; targeting PTMA might be a promising strategy for OSF treatment.

## MATERIALS AND METHODS

### Serum and oral submucosa sample

The 7 OSF and 3 healthy control serum samples were collected for autoantigen profiling by microarray. For the ELISA assay, we collected 50 serum samples including 30 OSF samples and 20 healthy control samples. For the QPCR assay, we collected 16 healthy control oral submucosa samples and 52 oral submucosa samples from patients with OSF. All the serum and submucosa smaples were collected at the Xiangya Hospital, Central South University. All of the enrolled OSF patients and healthy volunteers signed informed consent forms. The study was approved by the Ethic Committee of Xiangya Hospital, Central South University. Healthy control volunteers are without any autoimmune diseases.

### Human proteome microarrays assay

The human proteome microarray used in the first phase of this study was composed of about 19,000 unique human full-length proteins (HuProt™) and was purchased from Beijing Protein Innovation Co. Ltd (Beijing, China). Each of the recombinant human proteins was printed in duplicate, as were the control probes (printing buffer, human IgG, etc.). The serum assay on proteome microarrays were carried out as previous described [26]. In brief, after blocking, the 1:1,000 diluted patient sera were incubated with the microarrays. Second, after washing away the sera, the 1:1,000 diluted Cy5-labeled goat anti-human IgG antibody was applied. After the microarray was scanned and probes’ signal intensities were acquired, positive calling in each microarray was conducted according to the procedure previously described by Hu et al. [27]. OSF-specific autoantigen candidates were identified according to the following criteria: (a) Fisher’s exact test on a positive incidence showing statistical significance between OSF and control samples (*P* < 0.05) or (b) positive rate above 30%.

### Construction and purification of the OSF-associated autoantigen protein with GST or His tag

28 OSF-associated autoantigen genes were amplified from human cDNA by PCR. the sequences encoding 19 proteins CCDC97, CLUAP1, GAGE8, NOL3, RWDD1, ZNF428, UBQLN1, SET, CALR, FAM188A, UBL7, NASP, GAGE1, GAGE10, NACA, PAF1, HLA-DMB, AAMP and ANP32 were cloned into the pGEX-6P-1 vector which contained opening reading frame (ORF) of GST tag at the N-terminus. The gene encoding 9 proteins DTNBP1, NFU1, PTMA, PTMS, GORASP1, CHAC2, TERFZIP, MYLK and RBPJ were cloned into pET22b (+) vector which contained ORF of 6 × His tag. Those plasmids were transformed into Escherichia coli BL21 (DE3) respectively. The transformed cells were cultured in Luria-Bertani (LB) broth at 37 °C till OD_600_ = 0.8 and then induced with 0.5 mM IPTG. The expression of protein was carried out at 37 °C for 5 hours. The induced cells were harvested by centrifugation at 14000 g and resuspended in 20 mL lysis buffer (1% Triton X-100, 1 mM PMSF, 1×PBS, pH 7.6), then lysed by sonication. The lysate was centrifuged at 14000 g and 4 °C for 20 min to remove precipitate. The supernatant from His tage group was loaded on Ni^2+^-NTA column (GE Health, chelating sepharose, 17-0575-02). The column was balanced with 20 mL lysis buffer and washed with 20 mL lysis buffer containing 50 mM imidazol. The supernatant from GST-tag group was loaded on GSTrap FF column (Amersham Biosciences, USA). The column was balanced with 20 mL lysis buffer and washed with 20 mL lysis buffer containing 20 mM Tris-HCl, 20 mM GSH, 1mM DDT and 1 mM EDTA. The fractions containing the recombinant protein were collected and dialyzed against reaction buffer containing 20 mM Tris, 100 mM NaCl, 1 mM DTT and 1 mM EDTA at 4 °C (Supplementary Figure 1).

### ELISA

ELISA was performed as previous described with modification [28, 29]. Purified recombinant proteins were coated onto 96-well plates at 4 °C overnight. Nonspecific binding was blocked by incubating with 200 μl of PBS plus Tween 20 containing 1% BSA/well at 37 °C 1 h. The wells were incubated with human sera (1:100) at 37 °C for 1 h and then washed three times with PBS plus Tween 20. Subsequently, 100 μl of horseradish peroxidase-labeled mouse anti-human IgG monoclonal antibody (1:1,000; Beijing Protein Innovation Co. Ltd.) was added to each well. After three washes with PBS plus Tween 20, 100μl of tetramethybenzidine substrate solution (Sigma-Aldrich) was added and incubated for 15 min at room temperature. The reaction was terminated by addition of 50μl of 2 N H_2_SO_4_/well, and immunoreactivity was measured by reading the *A*_450_ in a microplate reader (Bio-rad, CA, USA). The relative absorption value for a given sample was calculated by dividing the average absorption value of duplicate by means of duplicate reference samples in the same ELISA plate. We set *A*_450_ > 0.2 as the cutoff for positive hits.

### Quantitative real-time (Q)-PCR analysis

We extracted total RNA from oral submucosa tissues and cultured cells using Trizol reagent (Invitrogen, CA, USA). QPCR was carried performed using an AB7500 real-time PCR instrument (Applied Biosystems CA, USA). SYBR^®^Premix Ex Taq™ (TaKaRa Bio. Inc. Japan) was used to measure the expression of CALR, NOL3, AAMP, PTMA, PTMS, GORASP1, ZNF48, NASP, DTNBP1, TGFβ1, SMAD4 and Collagen I. β-actin was used as an endogenous control. 2^-ΔΔCT^ method was applied for data processing. The primers were listed in Supplementary Table 2.

### Western blot assays

Protein concentration was determined by the bicinchoninic acid (BCA) protein assay, and denatured proteins were separated in 10-15% SDS polyacrylamide gel electrophoresis and transferred onto PVDF membranes. Nonspecific binding was blocked with 5% milk in TBST buffer for 2 h, followed by incubation with primary antibodies (PTMA, Collagen I, α-MSA, MMP9, SMAD4 and β-actin, Santa Cruz, TX, USA) at 4°C overnight and secondary antibodies (mouse anti-rabbit and goat-anti-mouse secondary antibodies, Santa Cruz, USA) at room temperature for 2 h. Blots were visualized using ECL detection reagents.

### Immunochemistry

For detection the expression of PTMA in oral submucosa tissue samples, immunochemistry was performed as previous described [30]. The oral submucosa tissue samples were fixed in 10% formalin and embedded in paraffin, then cut into 4 μm-thick tissue section. The sections were dewaxed and washed three times with PBS. After treated with endogenous peroxidase in 3% H_2_O_2_ for 20 min, each section was incubated in 10% normal goat serum for non-specific inhibition (Beyotime, China) for 30 min at 37°C and then incubated with PTMA antibody, overnight at 4°C. The sections were washed three times with PBS and were incubated with a secondary antibody linked with HRP for 30 min at 37°C. Subsequently, the sections were incubated in DAB reagent (Beyotime, China) and subsequently stained with hematoxylin (Beyotime, China). The sections were observed by microscope (Olympus, Tokyo, Japan).

### The protein-protein interactions (PPIs) network construction [31]

We obtained PPIs from public databases including STRING, BioGRID, IntAct and MINT, for the exploration of protein–protein interactions. Those most reliable PPIs were selected and used for the network construction that consists of protein PTMA, TGFβ1 and SMAD4 along with their direct sharing PPI neighbors and the interactions between these proteins. The network was illustrated using Cytoscape software (version 3.0).

### Cell culture and transfection

Primary human oral submucosal fibroblasts were isolated from normal oral submucosal tissue using tissue explants method as previous described [32]. Briefly, oral submucosa samples were washed with PBS and cut into small pieces. Then, the tissue pieces were seeded in cell culture flasks containing DMEM/F12 medium (GIBCO, USA) supplemented with 10% fetal calf serum (GIBCO, USA), 100 units/ml penicillin and 100 μg/ ml streptomycin and incubated with at 37 °C in a humidified incubator of 5% CO_2_ (Sanyo, Japan). The culture medium was replaced every 3 days. Subculture was performed when cells reached 80% confluence.

For TGFβ1 treatment, TGFβ1 (R&D Systerm, USA) were firstly dissolved in DMSO (Sigma, USA) and further diluted to 0, 2, 4, 6, 8, 10 ng/ml, respectively, the final DMSO concentration was less than 0.1% (v/v). Cells were treated with different concentration of TGFβ1 for 24h or 6 ng/ml TGFβ1 for 24, 48, 72 or 96h.

Si-PTMA (GenePharma, Shanghai, China) was transfected into target cells with siRNA-Mate transfection reagent (GenePharma) to achieve PTMA knockdown. PcDNA-PTMA vector (Genechem, Shanghai, China) was transfected into target cells with lipo2000 (Invitrogen, USA) to achieve PTMA overexpression. 48h after transfection, cells were harvested for western blotting assays.

### Cell viability assays

Cell Counting Kit-8 (CCK-8) (Beyotime, Hangzhou, China) was used to measure cell viability. We seeded 0.5×10^4^ cells in each 96-well plate for 24 h, transfected them with the indicated siRNA, and further incubated cells for 1, 2, 3, 4, 5 days, respectively. At 1 h before the endpoint of incubation we added 10 μl CCK-8 reagents to each well. A microplate reader was used to determine OD_490nm_ value in each well.

### Immunofluorescence

For the detection of Collagen I and α-SMA expression in primary human oral submucosal fibroblasts, cells (1×10^5^ per well) were seeded in 6-well glass-bottomed plate. After the cells were treated with 6 ng/ml TGβ1 for 48 h, they were fixed in 4% paraformaldehyde for 30 min and then permeabilized with 0.2% Triton X-100 for 15 min. Non-specific binding sites were blocked with 1% BSA in PBS for 2h. Then, the cells were treated with primary antibody specific to Collagen I and α-SMA (1:200, diluted in 1% BSA, respectively) overnight at 4 °C. Thereafter, the cells were incubated with FITC-conjugated secondary antibody (Beyotime, China) for 1 h at dark. The images were acquired using a fluorescence microscope (Nikon, Japan). The green fluorescence indicated Collagen I and α-SMA expression.

### Statistics

All statistical analysis was performed with SPSS 17.0 software. P values were calculated with Pearson’s Chi-square, Fisher’s exact, Spearman’s rank correlation analysis or one-way ANOVA tests. A *P* value less than 0.05 was considered to be statistically significant

## SUPPLEMENTARY MATERIALS FIGURE AND TABLES




