# Supplementary material for: PTMA, a new identified autoantigen for oral submucous fibrosis, regulates oral submucous fibroblast proliferation and extracellular matrix
Source: Oncotarget. 2017 Aug 24;8(43):74806–19. doi: 10.18632/oncotarget.20419 (PMC5650380; doi:10.18632/oncotarget.20419)
Supplement: Supplementary file 1 [file oncotarget-08-74806-s001.pdf]

PTMA, a new identified autoantigen for oral submucous fibrosis, regulates oral submucous fibroblast proliferation and extracellular matrix

SUPPLEMENTARY MATERIALS

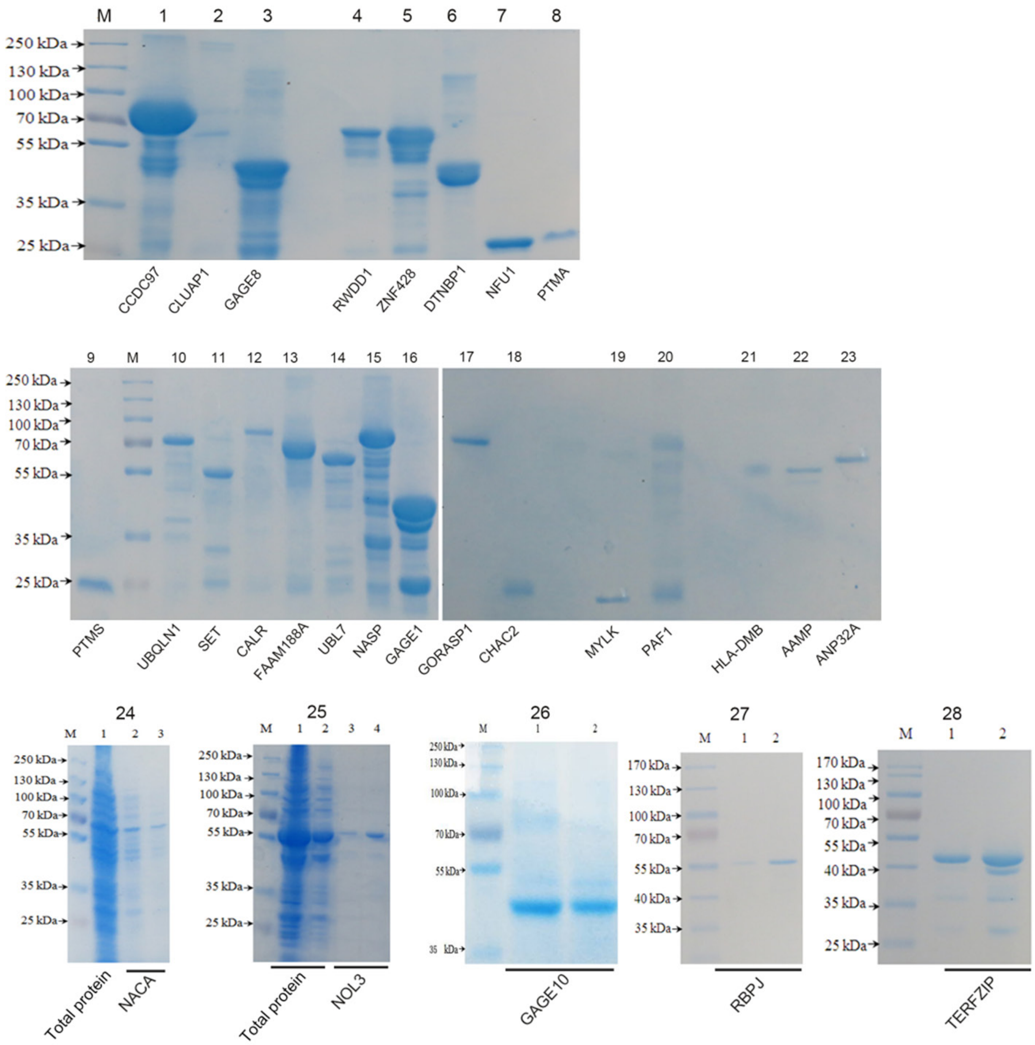

Supplementary Figure 1: SDS-PAGE analysis of the 28 prokaryotic expressed and purified OSF-associated autoantigen proteins.

Supplementary Table 1: Detail information for 45 identified OSF-associated autoantigens.

See Supplementary File 1

Supplementary Table 2: Primer sequence

Gene	Forward	Reverse
GORASP1	TGAAGGCACTACTGAAAGCCA	CCAACCACATAGTCTGTGTAGGG
CALR	CCTGCCGTCTACTTCAAGGAG	GAACTTGCCGGAACTGAGAAC
PTMA	TCAGACGCAGCCGTAGACA	GCATTCCCGTTAGCAGGGG
NOL3	GACCGCAGCTATGACCCTC	CTCCGGTTCAGCCTCTTTAGA
AAMP	CGAGGTGGTAGAACTTGATCCC	GATGCTGAGTGCAATGCAAAG
ZNF48	TGATTGCGGCAAGAGGTTTGT	GCGGAGGTGTTTGACTCGG
NASP	AGATTGGGAACCTAGAGCTTGC	ACTTCTCCGAGTTTAAGATGTGC
DTNBP1	GAGCTGGTGGATAGCGAGG	AGTCTGCGATTAAAGCTGGGA
TGFβ1	GGCCAGATCCTGTCCAAGC	GTGGGTTTCCACCATTAGCAC
SMAD4	CTCATGTGATCTATGCCCGTC	AGGTGATACAACTCGTTCGTAGT
Collagen I	GAGGGCCAAGACGAAGACATC	CAGATCACGTCATCGCACAAC
β-actin	CATGTACGTTGCTATCCAGGC	CTCCTTAATGTCACGCACGAT
