# Supplementary material for: PTMA, a new identified autoantigen for oral submucous fibrosis, regulates oral submucous fibroblast proliferation and extracellular matrix
Source: Oncotarget. 2017 Aug 24;8(43):74806–19. doi: 10.18632/oncotarget.20419 (PMC5650380; doi:10.18632/oncotarget.20419)
Supplement: Supplementary file 2 [file oncotarget-08-74806-s002.docx]

**Supplementary Table 1: Detail information for 45 identified OSF-associated autoantigens**

| **Accession** | **Description** |  | **Size** |
| --- | --- | --- | --- |
| XP_939608 | PREDICTED: hypothetical protein LOC55036 isoform 7 [Homo sapiens] | | 8 KB |
| NM_001098413 | Homo sapiens G antigen 10 (GAGE10), mRNA | | 14 KB |
| NM_001040663 | Homo sapiens G antigen 1 (GAGE1), transcript variant 2, mRNA | | 19 KB |
| NM_001009615 | Homo sapiens SPANX family, member N2 (SPANXN2), mRNA | | 12 KB |
| NM_001009609 | Homo sapiens SPANX-N3 protein (SPANX-N3), mRNA | | 13 KB |
| NM_001008708 | Homo sapiens ChaC, cation transport regulator homolog 2 (E. coli) (CHAC2), mRNA | | 14 KB |
| NM_001002756 | Homo sapiens NFU1 iron-sulfur cluster scaffold homolog (S. cerevisiae) (NFU1), nuclear gene encoding mitochondrial protein, transcript variant 3, mRNA | | 24 KB |
| NM_183041 | Homo sapiens dystrobrevin binding protein 1 (DTNBP1), transcript variant 3, mRNA | | 31 KB |
| NM_182498 | Homo sapiens zinc finger protein 428 (ZNF428), mRNA | | 18 KB |
| NM_153757 | Homo sapiens nucleosome assembly protein 1-like 5 (NAP1L5), mRNA | | 20 KB |
| NM_152298 | Homo sapiens nuclear autoantigenic sperm protein (histone-binding) (NASP), transcript variant 3, mRNA | | 37 KB |
| NM_152296 | Homo sapiens ATPase, Na+/K+ transporting, alpha 3 polypeptide (ATP1A3), mRNA | | 48 KB |
| NM_058163 | Homo sapiens TSR2, 20S rRNA accumulation, homolog (S. cerevisiae) (TSR2), mRNA | | 19 KB |
| NM_053031 | Homo sapiens myosin light chain kinase (MYLK), transcript variant 7, mRNA | | 31 KB |
| NM_052957 | Homo sapiens acidic repeat containing (ACRC), mRNA | | 12 KB |
| NM_052848 | Homo sapiens coiled-coil domain containing 97 (CCDC97), mRNA | | 18 KB |
| NM_032907 | Homo sapiens ubiquitin-like 7 (bone marrow stromal cell-derived) (UBL7), transcript variant 1, mRNA | | 21 KB |
| NM_031899 | Homo sapiens golgi reassembly stacking protein 1, 65kDa (GORASP1), mRNA | | 34 KB |
| NM_024948 | Homo sapiens family with sequence similarity 188, member A (FAM188A), mRNA | | 28 KB |
| NM_024793 | Homo sapiens clusterin associated protein 1 (CLUAP1), transcript variant 2, mRNA | | 19 KB |
| NM_019088 | Homo sapiens Paf1, RNA polymerase II associated factor, homolog (S. cerevisiae) (PAF1), mRNA | | 31 KB |
| NM_018975 | Homo sapiens telomeric repeat binding factor 2, interacting protein (TERF2IP), mRNA | | 25 KB |
| NM_016449 | Homo sapiens chromosome 22 open reading frame 43 (C22orf43), mRNA | | 19 KB |
| NM_015952 | Homo sapiens RWD domain containing 1 (RWDD1), transcript variant 1, mRNA | | 27 KB |
| NM_015874 | Homo sapiens recombination signal binding protein for immunoglobulin kappa J region (RBPJ), transcript variant 2, mRNA | | 34 KB |
| NM_012196 | Homo sapiens G antigen 8 (GAGE8), mRNA | | 32 KB |
| NM_005594 | Homo sapiens nascent-polypeptide-associated complex alpha polypeptide (NACA), mRNA | | 20 KB |
| NM_004343 | Homo sapiens calreticulin (CALR), mRNA | | 30 KB |
| NM_003946 | Homo sapiens nucleolar protein 3 (apoptosis repressor with CARD domain) (NOL3), mRNA | | 24 KB |
| NM_003011 | Homo sapiens SET translocation (myeloid leukemia-associated) (SET), mRNA | | 27 KB |
| NM_002824 | Homo sapiens parathymosin (PTMS), mRNA | | 26 KB |
| NM_002823 | Homo sapiens prothymosin, alpha (gene sequence 28) (PTMA), mRNA | | 20 KB |
| NM_002482 | Homo sapiens nuclear autoantigenic sperm protein (histone-binding) (NASP), transcript variant 2, mRNA | | 39 KB |
| NM_002118 | Homo sapiens major histocompatibility complex, class II, DM beta (HLA-DMB), mRNA | | 29 KB |
| NM_001087 | Homo sapiens angio-associated, migratory cell protein (AAMP), mRNA | | 37 KB |
| BC098149 | Homo sapiens variable charge, X-linked 3A, mRNA (cDNA clone MGC:118976 IMAGE:40002644), complete cds | | 16 KB |
| BC090928 | Homo sapiens SLAM family member 6, mRNA (cDNA clone IMAGE:3066534), with apparent retained intron | | 17 KB |
| BC070336 | Homo sapiens immunoglobulin kappa constant, mRNA (cDNA clone IMAGE:30330282) | | 21 KB |
| BC062732 | Homo sapiens immunoglobulin kappa constant, mRNA (cDNA clone IMAGE:30351013) | | 22 KB |
| BC036743 | Homo sapiens ubiquilin 3, mRNA (cDNA clone MGC:44847 IMAGE:5167911), complete cds | | 19 KB |
| BC032749 | Homo sapiens SET nuclear oncogene, mRNA (cDNA clone MGC:45315 IMAGE:5587291), complete cds | | 19 KB |
| BC014122 | Homo sapiens angio-associated, migratory cell protein, mRNA (cDNA clone IMAGE:4552582), containing frame-shift errors | | 16 KB |
| BC007200 | Homo sapiens acidic (leucine-rich) nuclear phosphoprotein 32 family, member A, mRNA (cDNA clone MGC:12667 IMAGE:3677623), complete cds | | 16 KB |
| BC000267 | Homo sapiens GC-rich promoter binding protein 1, mRNA (cDNA clone IMAGE:3357748), complete cds | | 17 KB |
| AK314899(NOL3) | Homo sapiens cDNA, FLJ95803, Homo sapiens nucleolar protein 3 (apoptosis repressor with CARD domain) (NOL3), mRNA | | 10 KB |
